# Endocardite Infecciosa: Um Desafio Constante?

**DOI:** 10.36660/abc.20250665

**Published:** 2026-06-10

**Authors:** Catarina Gregório, Ana Beatriz Garcia, Susana Gonçalves, Nuno Guerra, Fausto J. Pinto, Catarina Sousa

**Affiliations:** 1 Universidade de Lisboa Faculdade de Medicina Heart and Vessels Department, Hospital de Santa Maria Lisbon Portugal Heart and Vessels Department, Hospital de Santa Maria (ULSSM), CAML, CCUL@RISE, Faculdade de Medicina, Universidade de Lisboa, Lisbon – Portugal

**Keywords:** Endocardite de Prótese Valvular, Endocardite Infecciosa Recorrente, Pseudoaneurisma, Imagem Multimodal, Cirurgia Cardíaca

## Introdução

A endocardite de válvula protética (EVP) é uma condição incomum, porém potencialmente fatal. Este relato descreve um caso complexo de EVP recorrente com complicações paravalvulares que exigiu múltiplas intervenções cirúrgicas.

## Descrição

Um homem afrodescendente de 38 anos com doença cardíaca valvular reumática foi submetido a substituição de válvula aórtica por uma prótese mecânica Sorin de 25 mm em novembro de 2017 por insuficiência aórtica grave. O ecocardiograma pré-operatório documentou uma válvula aórtica tricúspide com espessamento dos folhetos, *doming* e leve fibrocalcificação, compatíveis com envolvimento reumático, e insuficiência grave (Figura Suplementar 1). A válvula mitral apresentava alterações reumáticas leves (espessamento dos folhetos e *doming* do folheto anterior) com insuficiência leve e sem impacto funcional à data.

O paciente teve alta medicado com varfarina 5 mg, furosemida 40 mg e enalapril 5 mg. Não tinha história de uso de drogas intravenosas, terapêutica com corticosteroides ou imunossupressão.

**Figura 1:**
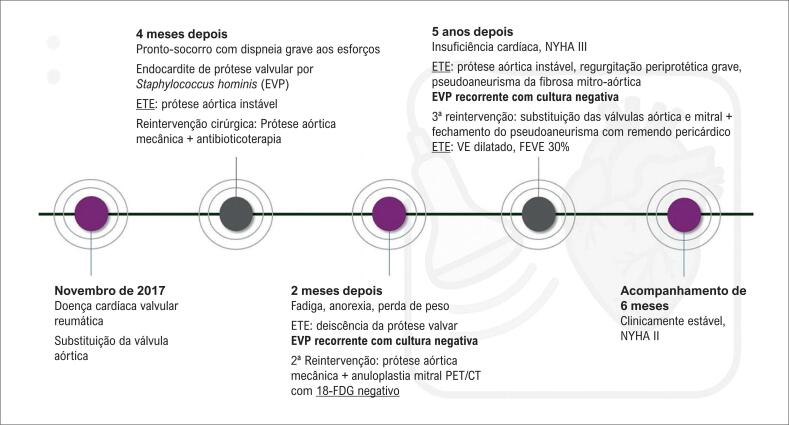
Cronologia da Evolução Clínica. Cronologia da apresentação e progressão clínica do paciente, destacando a complexidade e os desafios envolvidos no manejo da endocardite recorrente de válvula protética. ETE: ecocardiograma transesofágico; PET/CT: tomografia por emissão de pósitrons com tomografia computorizada; NYHA: New York Heart Association; VE: ventrículo esquerdo; FEVE: fração de ejeção do ventrículo esquerdo.

Quatro meses depois, o paciente foi admitido no pronto-socorro com dispneia de esforço (Figura 1). Ao exame, se apresentava afebril, com pressão arterial de 133/61 mmHg e frequência cardíaca de 89 bpm. A auscultação cardíaca revelou primeira bulha cardíaca mecânica e sopro sistólico na área aórtica, enquanto o exame pulmonar não apresentou alterações.

O ecocardiograma transesofágico (ETE) revelou deiscência da prótese valvular aórtica com movimento de báscula e envolvimento mitral reumático com regurgitação leve. Iniciou-se antibioticoterapia empírica com vancomicina e gentamicina. Os exames laboratoriais mostraram anemia normocítica (Hb 10,6 g/dL), elevação dos marcadores cardíacos (troponina T 45 ng/L e NT-proBNP 868 pg/mL) e proteína C-reativa baixa (0,56 mg/dL). As hemoculturas foram negativas. Não ocorreram eventos embólicos. A higiene oral e a saúde dentária eram boas, e nenhuma porta de entrada foi identificada.

Foi realizada uma nova cirurgia com implante de uma prótese aórtica mecânica Sorin de 23 mm; *Staphylococcus hominis* foi isolado da prótese; embora os estafilococos coagulase-negativos (ECN) possam representar contaminantes, os achados clínicos e de imagem corroboraram o diagnóstico de EVP verdadeira.

O paciente recebeu antibioticoterapia direcionada por 6 semanas. O seu pós-operatório foi complicado por bloqueio atrioventricular completo, que persistiu por seis dias após a cirurgia, levando à implantação de marca-passo bicameral. Após completar o ciclo de 6 semanas de antibioticoterapia, as hemoculturas repetidas permaneceram negativas e o ecocardiograma transtorácico documentou uma prótese bem posicionada.

Dois meses depois, o paciente retornou com fadiga, anorexia e perda ponderal não intencional (6 kg em duas semanas), permanecendo afebril. O ecocardiograma transtorácico, complementado por ETE, revelou regurgitação aórtica torrencial devido a uma nova deiscência de prótese e regurgitação mitral grave. Hemoculturas seriadas permaneceram negativas. A investigação extensa para infecções e doenças autoimunes — incluindo sorologias para *Coxiella, Bartonella e Brucella*, bem como um painel autoimune (anticorpos antinucleares, anti-DNA de dupla fita e anticorpos antifosfolipídicos [anticoagulante lúpico, anticardiolipina e anti-β2 glicoproteína]) — foi negativa. As culturas fúngicas também foram negativas. Suspeitou-se de endocardite protética recorrente e ele recebeu um ciclo de seis semanas de vancomicina, gentamicina e rifampicina.

Após completar o ciclo completo de antibióticos, com hemoculturas persistentemente negativas, ele foi submetido a uma terceira cirurgia: substituição da válvula aórtica por uma prótese mecânica St. Jude de 25 mm e anuloplastia mitral com um anel Edwards de 32 mm.

**Figura 2:**
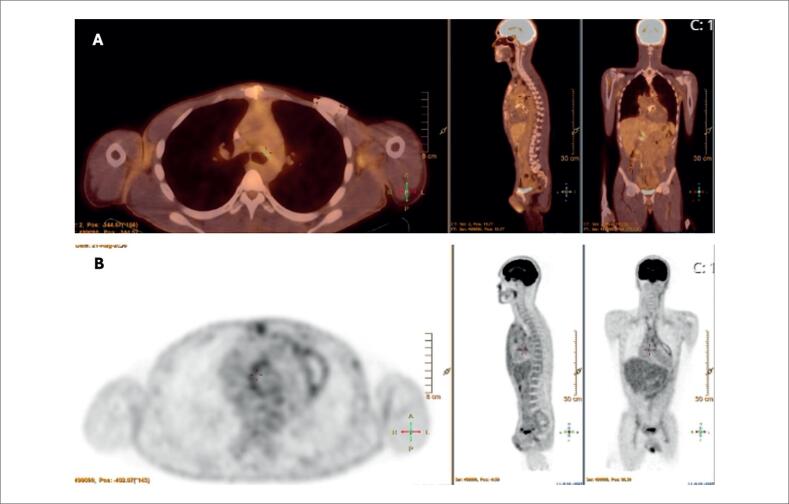
PET/CT com ^18^F-FDG pós-operatório do paciente. A - Ausência de hipermetabolismo focal anormal, confirmando a ausência de infecção residual da prótese valvular. B - Captação perivalvar difusa compatível com alterações pós-operatórias recentes.

O exame PET/CT com ^18^F-FDG pós-operatório demonstrou captação perivalvular difusa compatível com cirurgia recente, mas sem hipermetabolismo focal anormal, confirmando a ausência de infecção residual (Figuras 2A e 2B). O dispositivo de estimulação cardíaca e os eletrodos foram mantidos após discussão multidisciplinar (equipes de eletrofisiologia e infectologia), ponderando os riscos da extração do dispositivo contra a ausência de evidência de infecção. O ecocardiograma de acompanhamento mostrou boa função protética, *leak* paravalvular aórtico mínimo, regurgitação mitral leve e função sistólica ventricular esquerda preservada. Os níveis de NT-proBNP estavam baixos (79 pg/mL) e o paciente permaneceu clinicamente estável e assintomático (classe I da New York Heart Association – NYHA) por vários anos, sob acompanhamento ambulatorial regular com ecocardiografia transtorácica.

**Figura 3:**
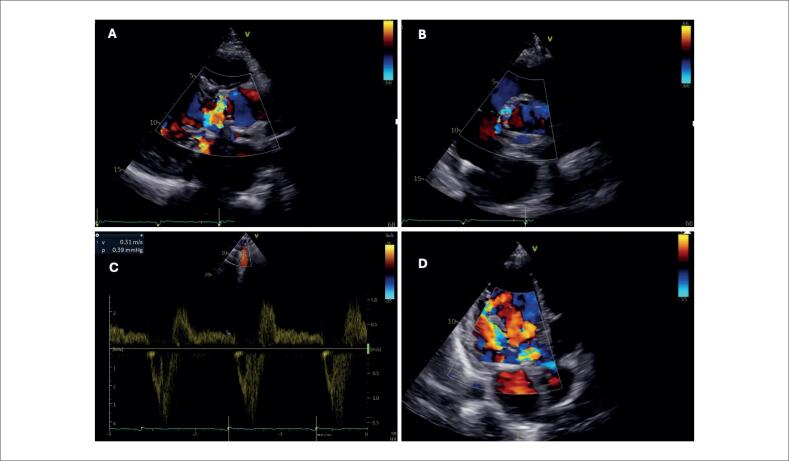
Ecocardiografia transtorácica demonstrando um jato regurgitante periprotético. A - Vista paraesternal de eixo longo. B - Vista de eixo curto. C - Doppler colorido mostrando inversão do fluxo holodiastólico no istmo aórtico, com velocidade terminal de 31 cm/s. D - Vista apical de três câmaras mostrando um jato regurgitante excêntrico que se estende por toda a cavidade ventricular esquerda.

**Figura 4:**
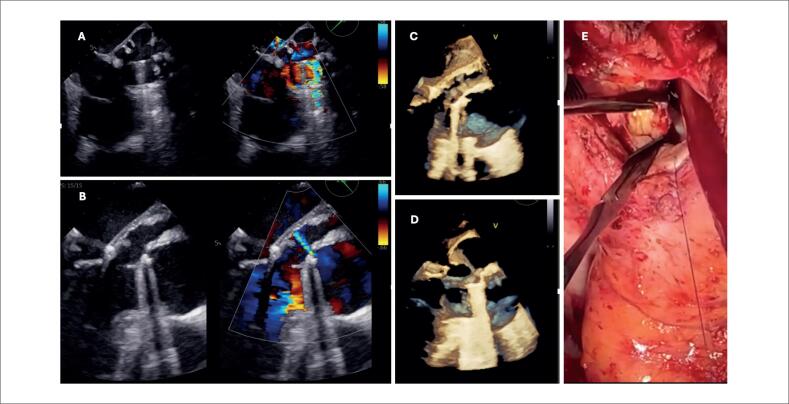
Ecocardiografia transesofágica e achados intraoperatórios. A - Vista em eixo curto da valva aórtica mostrando movimento de "balanço" da prótese, compatível com deiscência, e regurgitação periprotética grave entre as 7 e as 11 horas, envolvendo 25% da circunferência protética. B - Vista em eixo longo da aorta demonstrando um espaço perivalvar anecoico com fluxo Doppler colorido e expansão sistólica, compatível com um pseudoaneurisma periprotético (na fibrosa mitro-aórtica). C e D - Imagens 3D de ecocardiografia transesofágica delineando o pseudoaneurisma, estendendo-se cranialmente para a aorta ascendente e caudalmente para o ventrículo esquerdo. E - Imagem intraoperatória mostrando extenso desbridamento do trato de saída do ventrículo esquerdo.

Cinco anos depois, o paciente apresentou dispneia progressiva (NYHA III), ortopneia e congestão pulmonar. O ecocardiograma transtorácico mostrou uma prótese aórtica com funcionamento normal (gradiente médio de 22 mmHg, índice de velocidade Doppler (DVI) de 0,49, relação tempo de aceleração/tempo de ejeção de 0,42), mas com grave *leak* paravalvular (entre 7 e 11 horas, aproximadamente 30% da circunferência) e *rocking* da prótese (Figura 3). O ETE confirmou a presença de um pseudoaneurisma da fibrosa mitroaórtica contíguo à aorta ascendente e ao ventrículo esquerdo (Figuras 4A e 4B). O ETE tridimensional foi fundamental para delinear a extensão do pseudoaneurisma e orientar o planejamento cirúrgico (Figuras 4C e 4D). O ventrículo esquerdo estava gravemente dilatado, com fração de ejeção preservada (FEVE 59%), embora em um contexto de sobrecarga de volume significativa. A pressão sistólica da artéria pulmonar era de 31 mmHg e não foram detectadas vegetações ou abscessos (Vídeos Suplementares 1 e 2). As hemoculturas e as sorologias repetidas foram novamente negativas.

**Figura 5:**
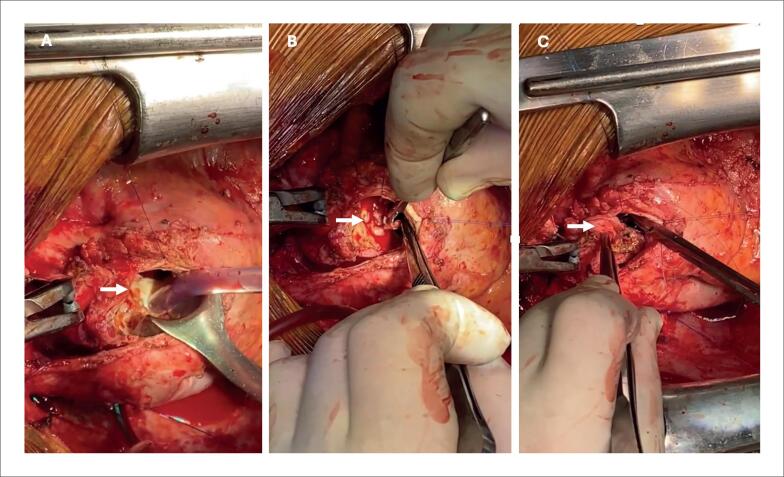
Imagens intraoperatórias mostrando a substituição simultânea das válvulas aórtica e mitral, juntamente com o fechamento de um pseudoaneurisma utilizando um remendo pericárdico autólogo. A – Envolvimento da fibrosa mitro-aórtica (seta); B – Desbridamento extenso do pseudoaneurisma na fibrosa mitro-aórtica (seta); C – Remendo pericárdico autólogo sendo suturado à fibrosa mitro-aórtica.

Diante da suspeita de EVP recorrente com complicação perivalvular, ele foi submetido a uma quarta cirurgia com substituição das válvulas aórtica (St. Jude Regent 23 mm) e mitral (St. Jude Master 29 mm), com fechamento do pseudoaneurisma utilizando um remendo pericárdico autólogo (Figura 4E e Figura 5). O ecocardiograma pós-operatório revelou dilatação grave do ventrículo esquerdo e fração de ejeção reduzida (FEVE 30%), regurgitação periprotética aórtica leve a moderada e regurgitação protética mitral leve. Foi iniciado tratamento medicamentoso para insuficiência cardíaca, conforme as diretrizes, e seu dispositivo foi atualizado para CRT-P. Após 6 meses de acompanhamento, ele permanece clinicamente estável em classe II da NYHA.

## Discussão

A EVP é uma complicação relativamente incomum, porém grave, representando 5–20%^1–3^ de todos os casos de endocardite infecciosa e associada a morbidade e mortalidade substanciais.^4^ Estafilococos, particularmente *Staphylococcus aureus* e ECN, são os patógenos mais frequentes na EVP precoce^4^ e estão consistentemente associados a um prognóstico adverso,³ envolvendo mais frequentemente a válvula aórtica.^4^ Infecções por ECN, como neste caso, estão particularmente relacionadas com complicações tardias, incluindo *leak* paravalvar, deiscência de prótese e formação de pseudoaneurisma.^5^

A apresentação inicial do nosso paciente foi indolente e atípica, permanecendo afebril com resposta inflamatória mínima e hemoculturas persistentemente negativas e nenhum fenômeno embólico. As culturas da válvula cirúrgica identificaram *Staphylococcus hominis*, ressaltando o valor diagnóstico da cultura intraoperatória da válvula em casos suspeitos com cultura negativa. Embora ECN como S. *hominis* possam ocasionalmente representar contaminação,^6^ a combinação de achados clínicos, de imagem (deiscência da prótese) e laboratoriais (por exemplo, anemia) corroborou um verdadeiro processo infeccioso.

Testes sorológicos extensivos, incluindo sorologias repetidas para *Coxiella, Bartonella* e *Brucella*, e testes autoimunes, também foram essenciais para excluir agentes infecciosos menos comuns^7^ e endocardite não infecciosa,^1^ que pode mimetizar endocardite com deiscência de prótese. A endocardite fúngica foi considerada improvável devido ao estado imunocompetente do paciente e à ausência de fatores de risco clássicos (como uso de drogas intravenosas ou presença de cateteres).^8^ Contudo, foram realizadas culturas fúngicas, com resultado negativo, confirmando uma etiologia bacteriana. Não foi identificada nenhuma porta de entrada clara e a saúde bucal foi documentada como boa.

Além dos desafios no diagnóstico microbiológico, a formação de biofilme é outro fator crucial na EVP. Os ECN e outros microrganismos de baixa virulência podem aderir ao material protético e formar um biofilme, no qual as bactérias persistem em estado metabolicamente inativo. Esse microambiente reduz a penetração e a eficácia dos antibióticos e contribui para resultados negativos nas culturas e complicações locais tardias, como vazamento paravalvar e envolvimento do anel valvar.^9^

Técnicas de diagnóstico molecular, incluindo PCR e sequenciamento de rRNA 16S em tecido valvular excisado, assim como a análise histopatológica podem aumentar a precisão diagnóstica em casos com cultura negativa.^10^ Essas técnicas permitem a detecção e identificação de microrganismos que podem ser não cultiváveis ou presentes em quantidades muito baixas.^10^ No entanto, esses métodos diagnósticos avançados não estavam disponíveis em nosso centro e, portanto, não foram realizados neste paciente, o que representa uma limitação potencial na identificação do patógeno causador; não obstante, eles constituem uma abordagem recomendada na avaliação de EVP com cultura negativa.^1^

O exame histopatológico das válvulas protéticas não foi realizado durante as reoperações; apenas a válvula aórtica nativa inicial foi examinada, confirmando o envolvimento reumático. Isso representa uma limitação, embora as análises moleculares e histopatológicas continuem sendo recomendadas na avaliação de endocardite protética com cultura negativa.

A imagem multimodal foi fundamental tanto no diagnóstico quanto no acompanhamento longitudinal, constituindo um critério diagnóstico modificado pela ESC para endocardite infecciosa.^1^ Embora a sensibilidade do ETE na endocardite protética seja menor do que na endocardite de válvula nativa,^11^ ele permanece essencial para a detecção de complicações protéticas. O ETE tridimensional proporcionou uma caracterização espacial detalhada do pseudoaneurisma da fibrosa mitroaórtica, melhorando a localização e auxiliando o planejamento cirúrgico além da imagem bidimensional convencional.^1,12^

O PET/CT com ^18^F-FDG pós-operatório excluiu infecção residual da prótese ou peri-anular e focos extracardíacos, reforçando seu papel complementar em casos complexos de endocardite protética. Notavelmente, o PET/CT com ^18^F-FDG é agora reconhecido como um importante critério diagnóstico na classificação de Duke modificada, refletindo sua crescente importância na prática clínica de rotina.^1^

O curso clínico do paciente foi complicado por deiscência protética recorrente, formação de pseudoaneurisma e remodelamento ventricular progressivo, exigindo múltiplas reoperações e terapia com dispositivo para insuficiência cardíaca. Tais eventos são bem documentados na endocardite protética e contribuem para o seu mau prognóstico, levando à insuficiência cardíaca avançada e aumento da mortalidade.^4^ Os fatores de risco relatados para recorrência incluem infecção estafilocócica (especialmente por ECN), próteses mecânicas, cirurgias cardíacas prévias e complicações estruturais locais, como pseudoaneurisma ou deiscência.^2,13,14^ Idade mais jovem, uso de drogas intravenosas ou infecção pelo HIV e técnica cirúrgica também foram identificados como preditores de reoperação.^13^ Embora a técnica cirúrgica inadequada possa contribuir para complicações recorrentes,^13,15^ todos os procedimentos neste caso foram realizados em um centro de cirurgia cardíaca de alto volume e com vasta experiência, por uma equipe cirúrgica experiente, ressaltando a gravidade da patologia subjacente e o valor de uma abordagem diagnóstica multidisciplinar e multimodal.

Embora a terapêutica antibiótica supressiva vitalícia tenha sido proposta em cenários selecionados de alto risco de endocardite infecciosa - particularmente quando o material infectado não pode ser totalmente removido - as evidências permanecem limitadas e seu papel na EVP permanece incerto devido à falta de estudos comparativos.^16,17^ Neste paciente, que não era imunossuprimido, avaliações clínicas, microbiológicas e de imagem seriadas demonstraram consistentemente a ausência de infecção residual, e as hemoculturas permaneceram consistentemente negativas. Em colaboração com especialistas em Doenças Infecciosas, se optou por não realizar terapia supressiva, visto que os benefícios teóricos não superavam os riscos, dada a ausência de evidência de infecção em curso.

Apesar desses desafios, o paciente permanece clinicamente estável sob vigilância contínua. Este caso ilustra que desfechos favoráveis em casos de endocardite protética recorrente dependem do reconhecimento clínico precoce, de exames de imagem multimodais abrangentes, de terapia antimicrobiana personalizada e de cirurgia oportuna dentro de uma abordagem multidisciplinar.

## Data Availability

Os conteúdos subjacentes ao texto da pesquisa estão contidos no manuscrito.

## *Material suplementar

*Material suplementar Para Figura Suplementar, por favor, clique aqui. Para Vídeo Suplementar 1, por favor, clique aqui. Para Vídeo Suplementar 2, por favor, clique aqui.
