# Supplementary material for: Endocardite Infecciosa: Um Desafio Constante?
Source: Arq Bras Cardiol. 2026 Jun 10;123(4):e20250665. [Article in Portuguese] doi: 10.36660/abc.20250665 (PMC13398857; doi:10.36660/abc.20250665)

Supplemental Figure

Figure 1 – Preoperative echocardiographic assessment. (A, B) Transthoracic echocardiography of the aortic valve in long-axis (A) and short-axis (B) views showing a tricuspid valve with thickened cusps, doming, and mild fibrocalcification, including a small calcific nodule on the non-coronary cusp, consistent with rheumatic involvement. (C) Mitral valve in long-axis view showing anterior leaflet doming and thickening, with mild regurgitation and no significant functional impact.

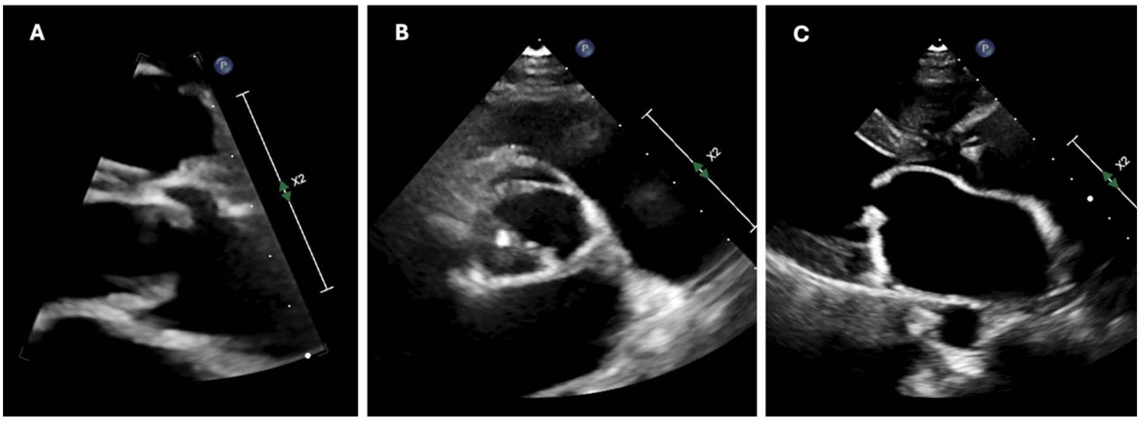

Supplement: *Material suplementar [file 0066-782X-abc-123-4-e20250665-Supp01.pdf]
