# Supplementary material for: Endocardite Infecciosa: Um Desafio Constante?
Source: Arq Bras Cardiol. 2026 Jun 10;123(4):e20250665. [Article in Portuguese] doi: 10.36660/abc.20250665 (PMC13398857; doi:10.36660/abc.20250665)
Supplement: Translation

# Translation: Infective Endocarditis: A Never-Ending Ordeal?

**DOI:** 10.36660/abc.20250665i

**Authors:** Catarina Gregório, Ana Beatriz Garcia, Susana Gonçalves, Nuno Guerra, Fausto J. Pinto, Catarina Sousa

**Affiliations:** 1 Lisbon Portugal Heart and Vessels Department, Hospital de Santa Maria (ULSSM), CAML, CCUL@RISE, Faculdade de Medicina, Universidade de Lisboa, Lisbon – Portugal

**Keywords:** Prosthetic Valve Endocarditis, Recurrent Infective Endocarditis, Pseudoaneurysm, Multimodality Imaging, Cardiac Surgery

## Introduction

Prosthetic valve endocarditis (PVE) is an uncommon but life-threatening condition. This report describes a challenging case of recurrent PVE with paravalvular complications requiring multiple surgical interventions.

## Description

A 38-year-old Black male with rheumatic valvular heart disease underwent aortic valve replacement with a 25-mm Sorin mechanical prosthesis in November 2017 due to severe aortic regurgitation. Preoperative echocardiography showed a tricuspid aortic valve with leaflet thickening, doming, and mild fibrocalcification, consistent with rheumatic involvement, and severe regurgitation (Supplemental Figure 1). The mitral valve exhibited mild rheumatic changes (leaflet thickening and anterior leaflet doming) with mild regurgitation and no functional impact at that time.

He was discharged on warfarin 5 mg, furosemide 40 mg, and enalapril 5 mg. He had no history of intravenous drug use, corticosteroid therapy, or immunosuppression.

Four months later, he was admitted to the emergency department with severe exertional dyspnea (Figure 1). On examination, he was afebrile with a blood pressure of 133/61 mmHg and a heart rate of 89 bpm. Cardiac auscultation revealed a mechanical first heart sound and a systolic murmur over the aortic area, while pulmonary examination was unremarkable.

**Figure 1:** Timeline of Clinical Course. Timeline of the patient's presentation and clinical progression, highlighting the complexity and challenges involved in the management of recurrent prosthetic valve endocarditis. ED: emergency department; TEE: transoesophageal echocardiography; PET/CT: positron emission tomography/computed tomography; NYHA: New York Heart Association; LV: left ventricle; LVEF: left ventricular ejection fraction.

Transoesophageal echocardiography (TEE) revealed prosthetic aortic valve dehiscence with rocking motion and rheumatic mitral involvement with mild regurgitation. He was started on empiric antibiotic therapy with vancomycin and gentamicin. Laboratory tests showed normocytic anemia (Hb 10.6 g/dL), elevated cardiac biomarkers (troponin T 45 ng/L and NT-proBNP 868 pg/mL), and low C-reactive protein (0.56 mg/dL). Blood cultures were negative. No embolic events occurred. Oral hygiene and dental health were good, and no portal of entry was identified.

Redo surgery with implantation of a 23-mm Sorin mechanical aortic prosthesis was performed; *Staphylococcus hominis* was isolated from the prosthesis; although coagulase-negative staphylococci (CoNS) may represent contaminants, clinical and imaging findings supported a true prosthetic valve endocarditis (PVE). The patient received targeted antibiotics for 6 weeks. His postoperative course was complicated by complete atrioventricular block, which persisted six days after surgery, prompting dual-chamber pacemaker implantation. After completing the 6-week antibiotic course, repeat blood cultures remained negative, and transthoracic echocardiography showed a well-seated prosthesis.

Two months later, he returned with fatigue, anorexia, and unintentional weight loss (6 kg in two weeks), remaining afebrile. Transthoracic echocardiography, complemented by TEE, revealed torrential aortic regurgitation due to new prosthetic dehiscence and severe mitral regurgitation. Serial blood cultures remained negative. Extended infectious and autoimmune screening — including serologies for *Coxiella*, *Bartonella*, and *Brucella*, as well as an autoimmune panel (antinuclear antibodies, anti-dsDNA, and antiphospholipid antibodies [lupus anticoagulant, anticardiolipin, and anti-β2 glycoprotein]) — were negative. Fungal cultures were also negative. Recurrent PVE was suspected, and he received a six-week course of vancomycin, gentamicin, and rifampicin.

After completing the full course of antibiotics, with persistently negative blood cultures, he underwent a third surgery: aortic valve replacement with a 25-mm St Jude mechanical prosthesis and mitral annuloplasty with a 32-mm Edwards ring.

Postoperative ^18^F-FDG PET/CT demonstrated diffuse perivalvular uptake consistent with recent surgery but no abnormal focal hypermetabolism, supporting the absence of residual infection (Figure 2A and 2B). The pacing device and leads were retained after multidisciplinary discussion (electrophysiology and infectious disease teams), balancing the risks of device extraction against the absence of evidence of device infection. Follow-up echocardiography showed good prosthetic function, minimal aortic paravalvular leak, mild mitral regurgitation, and preserved systolic function. NT-proBNP levels were low (79 pg/mL), and the patient remained clinically stable and asymptomatic (New York Heart Association – NYHA class I) for several years under regular outpatient follow-up with transthoracic echocardiography.

**Figure 2:** Postoperative ^18^F-FDG PET/CT of the patient. A - Absence of abnormal focal hypermetabolism, supporting no evidence of residual prosthetic valve infection. B - Diffuse perivalvular uptake consistent with recent postoperative changes.

Five years later, he presented with progressive dyspnea (NYHA III), orthopnea, and pulmonary congestion. Transthoracic echocardiography showed a normally functioning aortic prosthesis (mean gradient 22 mmHg, Doppler velocity index (DVI) 0.49, acceleration time/ejection time ratio 0.42) but a severe paravalvular leak (7–11 o'clock, ~30% of circumference) and prosthetic rocking (Figure 3). TEE confirmed the presence of a pseudoaneurysm of the mitro-aortic fibrosa contiguous with the ascending aorta and left ventricle (Figures 4A and 4B). Three-dimensional TEE was critical for delineating the extent of the pseudoaneurysm and guiding surgical planning (Figures 4C and 4D). The left ventricle was severely dilated with preserved ejection fraction (LVEF 59%), though in the setting of significant volume overload; pulmonary artery systolic pressure was 31 mmHg, and no vegetations or abscesses were detected (Supplemental Videos 1 and 2). Blood cultures and repeat serologies were again negative.

**Figure 3:** Transthoracic echocardiography demonstrating a periprosthetic regurgitant jet. A - Parasternal long-axis view. B - Short-axis view. C - Color Doppler showing holodiastolic flow reversal in the aortic isthmus, with a terminal velocity of 31 cm/s. D - Apical three-chamber view depicting an eccentric regurgitant jet extending throughout the left ventricular cavity.

**Figure 4:** Transesophageal echocardiography and intraoperative findings. A - Short-axis view of the aortic valve showing "rocking motion" of the prosthesis, consistent with dehiscence, and severe periprosthetic regurgitation at the 7 to 11 o'clock position, involving 25% of the prosthetic circumference. B - Long-axis view of the aorta demonstrating a perivalvular echo-free space with color Doppler flow and systolic expansion, consistent with a periprosthetic pseudoaneurysm (at the mitral–aortic fibrosa). C and D - 3D TEE views delineating the pseudoaneurysm, extending cranially into the ascending aorta and caudally into the left ventricle. E - Intraoperative imaging showing extensive left ventricular outflow tract debridement.

Given the suspicion of recurrent PVE with perivalvular complication, he underwent a fourth surgery with replacement of both the aortic (St Jude Regent 23 mm) and mitral valves (St Jude Master 29 mm), with closure of the pseudoaneurysm using an autologous pericardial patch (Figure 4E and Figure 5). Postoperative echocardiography revealed a severely dilated left ventricle and reduced ejection fraction (LVEF 30%), mild-to-moderate aortic periprosthetic regurgitation, and mild mitral prosthetic regurgitation. Guideline-directed medical therapy for heart failure was initiated, and his device was upgraded to CRT-P. At 6-month follow-up, he remains clinically stable in NYHA class II.

**Figure 5:** Intraoperative images showing simultaneous replacement of the aortic and mitral valves, along with closure of a pseudoaneurysm using an autologous pericardial patch. A – Involvement of the mitro-aortic fibrosa (arrow); B – Extensive debridement of the pseudoaneurysm at the mitro-aortic fibrosa (arrow); C – Autologous pericardial patch being sutured to the mitro-aortic fibrosa.

## Discussion

PVE is a relatively uncommon but severe complication, accounting for 5–20%^1–3^ of all cases of infective endocarditis and associated with substantial morbidity and mortality.^4^ Staphylococci, particularly *Staphylococcus aureus* and coagulase-negative staphylococci (CoNS), are the most frequent pathogens in early PVE^4^ and are consistently associated with adverse prognosis,^3^ most often involving the aortic valve.^4^ CoNS infections, such as in this case, are particularly linked to late complications, including paravalvular leak, prosthetic dehiscence, and pseudoaneurysm formation.^5^

Our patient's initial presentation was indolent and atypical, remaining afebrile with minimal inflammatory response, persistently negative blood cultures, and no embolic phenomena. Surgical valve cultures identified *Staphylococcus hominis*, underscoring the diagnostic value of intraoperative valve culture in suspected culture-negative cases. Although CoNS such as *S. hominis* may occasionally represent contamination,^6^ the combination of clinical, imaging (prosthetic dehiscence), and laboratory findings (e.g., anemia) supported a true infective process.

Extensive serological testing, including repeat serologies for *Coxiella*, *Bartonella*, and *Brucella*, and autoimmune testing, was also essential to exclude less common infectious agents^7^ and autoimmune-mediated valvulitis,^1^ which may mimic endocarditis with prosthetic dehiscence. Fungal endocarditis was considered unlikely given the patient's immunocompetent status and absence of classical risk factors (such as intravenous drug use or indwelling catheters);^8^ nonetheless, fungal cultures were performed and were negative, supporting a bacterial etiology. No clear portal of entry was identified, and oral health was documented as good.

In addition to the challenges in microbiological diagnosis, the role of biofilm formation is another key factor in PVE. CoNS and other low-virulence organisms can adhere to prosthetic material and form a biofilm, within which bacteria persist in a metabolically inactive state. This microenvironment reduces antibiotic penetration and efficacy and contributes to culture-negative presentations and late local complications such as paravalvular leak and annular involvement.^9^

Molecular diagnostic techniques, including PCR and 16S rRNA sequencing on excised valve tissue, as well as histopathological analysis, can increase diagnostic accuracy in culture-negative cases.^10^ These techniques allow the detection and identification of microorganisms that may be nonculturable or present in very low numbers.^10^ However, these advanced diagnostic methods were not available at our center and were therefore not performed in this patient, representing a potential limitation in identifying the causative pathogen; nonetheless, they constitute a recommended approach in the evaluation of culture-negative PVE.^1^

Histopathological examination of the prosthetic valves was not performed during the reoperations; only the initial native aortic valve was examined, confirming rheumatic involvement. This represents a limitation, while molecular and histopathological analyses remain recommended in the evaluation of culture-negative PVE.

Multimodality imaging was pivotal in both diagnosis and longitudinal management, constituting an ESC-modified diagnostic criterion for infective endocarditis.^1^ Although the sensitivity of TEE in PVE is lower than in native valve endocarditis,^11^ it remains essential for detecting prosthetic complications. Three-dimensional TEE provided detailed spatial characterization of the pseudoaneurysm of the mitro-aortic fibrosa, improving localization and aiding surgical planning beyond conventional two-dimensional imaging.^1,12^

Postoperative ^18^F-FDG PET/CT excluded residual prosthetic or peri-annular infection and extracardiac foci, reinforcing its complementary role in complex PVE. Notably, ^18^F-FDG PET/CT is now recognized as a major diagnostic criterion in the modified Duke classification, reflecting its growing importance in routine practice.^1^

The patient's clinical course was complicated by recurrent prosthetic dehiscence, pseudoaneurysm formation, and progressive ventricular remodeling requiring multiple redo surgeries and device therapy for heart failure. Such events are well documented in PVE and contribute to its poor prognosis, leading to advanced heart failure and increased mortality.^4^ Reported risk factors for recurrence include staphylococcal infection (especially CoNS), mechanical prostheses, prior cardiac surgeries, and local structural complications such as pseudoaneurysm or dehiscence.^2,13,14^ Younger age, intravenous drug use or HIV infection, and surgical technique have also been identified as predictors of reoperation.^13^ While inadequate surgical technique may contribute to recurrent complications,^13,15^ all procedures in this case were performed at a high-volume, experienced cardiac surgery center by an experienced surgical team, underscoring the severity of the underlying pathology and the value of a multidisciplinary, multimodal diagnostic approach.

Although lifelong suppressive antibiotic therapy has been proposed in selected high-risk scenarios of infective endocarditis—particularly when infected material cannot be fully removed—evidence remains limited, and its role in PVE remains uncertain due to the lack of comparative studies.^16,17^ In this patient, who was not immunosuppressed, serial clinical, microbiological, and imaging assessments consistently demonstrated the absence of residual infection, and blood cultures remained consistently negative. In collaboration with Infectious Disease specialists, suppressive therapy was not pursued, as theoretical benefits did not outweigh the risks, given the absence of evidence of ongoing infection.

Despite these challenges, the patient currently remains clinically stable under continued surveillance. This case illustrates that favorable outcomes in recurrent PVE depend on prompt clinical recognition, comprehensive multimodal imaging, tailored antimicrobial therapy, and timely surgery within a multidisciplinary framework.

## Data Availability

The underlying content of the research text is contained within the manuscript.

## *Supplemental Materials

*Supplemental Materials For Supplementary Figure, please click here. For Supplementary Video 1, please click here. For Supplementary Video 2, please click here.
